# Challenges and needs in dementia care: people with dementia and family caregivers’ experiences from diagnosis to follow-up in Swedish primary care: a qualitative study

**DOI:** 10.1080/02813432.2025.2529414

**Published:** 2025-07-12

**Authors:** Monica Bergqvist, Pia Bastholm-Rahmner, Karin Modig, Katharina Schmidt-Mende

**Affiliations:** ^a^ Academic Primary Health Care Centre, Region Stockholm, Stockholm, Sweden; ^b^ Department of Neurobiology, Care Sciences and Society, Division of Nursing, Karolinska Institutet, Stockholm, Sweden; ^c^ Department of Laboratory Medicine, Division of Clinical Pharmacology, Karolinska Institutet, Stockholm, Sweden; ^d^ Institute of Environmental Medicine, Unit of Epidemiology, Karolinska Institutet, Stockholm, Sweden; ^e^ Department of Neurobiology and Care Sciences and Society, Division of Family Medicine and Primary Care, Karolinska Institutet, Stockholm, Sweden

**Keywords:** Dementia, family caregivers, interviews, older people, primary care, thematic analysis

## Abstract

**Objective:**

Primary care plays a central role in diagnosing dementia and coordinating and providing care. This study explores how older people with dementia and their family caregivers experience the process from diagnosis to follow-up in primary care, what support is needed, what works well and what could be improved.

**Design:**

Semi-structured interviews were conducted with four people with dementia and 11 family caregivers in Stockholm, Sweden. Data were analyzed with inductive thematic analysis.

**Results:**

Four themes were identified: *Seamless assessment – from primary care to memory clinic*, describes the transition from primary care to memory clinics, where empathy and professionalism are crucial to prevent feelings of failure. *After diagnosis – a no man’s land*, highlights a communication gap between memory clinics and primary care that leaves informants feeling abandoned and causes emotional distress and isolation. *Follow-up in a deficient primary care*, underscores the importance of having ‘a single point of entry’ for coordinated support and a primary care provider with expertise in dementia. *Embracing life with the diagnosis*, emphasizes the need for guidance from a dedicated dementia team to manage progression.

**Conclusion:**

People with dementia and their caregivers felt that primary care often failed to meet their needs due to limited accessibility, poor coordination, and a lack of dementia-specific expertise. Strengthening the role of general practitioners, introducing liaison nurses, and enhancing collaboration with municipal services could improve continuity, navigation, and support. These findings underscore the need for Swedish health care policy to promote sustainable, person-centered dementia care models within primary care.

- Primary care should move from a one-size-fits-all approach to dementia care to targeted and more tailored interventions that take into account the needs of different individuals.
- An integrated care model is needed, where memory clinics, primary care and municipalities work together to support people with dementia and their families.
- People with dementia emphasize tailored support, especially in the early stages of the disease, to engage in meaningful social activities.

## Introduction

Globally, more than 50 million people live with dementia, of which approximately 150,000 are in Sweden [1]. This figure is expected to rise, putting great pressure on health care systems, family caregivers, and society at large [2]. The pathway from symptoms to diagnosis often involves a long process of observing, evaluating, and dismissing symptoms as normal, until the need for help becomes impossible to ignore [3–5]. Help-seeking for dementia symptoms is usually initiated by a caregiver or a close family member of the person with symptoms [3,4], and primary care (PC) is often the first point of contact [6,7].

According to the Swedish National Board of Health and Welfare, PC plays a key role both in the diagnostic process and in the provision and coordination of care for older people with dementia [8]. The assessment of suspected dementia often involves multiple health care professionals working in PC and outpatient specialist services, and it includes several steps (see Box 1). Furthermore, a person-centered, coherent care pathway for the assessment of cognitive impairment and suspected dementia should be accessible in each practice. Yet, family members and people with dementia often describe dementia care and community services as fragmented, uncoordinated, and difficult to navigate [6,9,10]. In addition, they report a lack of dementia care expertise among PC professionals and insufficient information and support [6,11–13]. At the same time, many family caregivers are unaware of their right to support services - such as home care, day care, and support groups - or have not been offered such help [7]. In Sweden, access to support also varies across municipalities and regions, leaving many caregivers lacking important information, knowledge, and resources [8].

There are limited studies on dementia care in PC from the Nordic countries. A Swedish qualitative study interviewing spouses caring for partners with dementia highlighted their need for individualized education and guidance to navigate the complexities of caregiving [13]. However, many felt that health care professionals lacked the expertise to provide support beyond clinical care. The standardized support model focused mainly on practical assistance and did not sufficiently address the emotional and relational needs of the family caregivers [13]. Similarly, a Finnish study involving individuals with memory disorders and their family caregivers found that they are often in a disadvantaged position, lacking influence over decisions related to health and social care [14]. Such decisions are frequently driven by structural factors rather than the preferences of the patient or principles of person-centered care.

Given the increasing number of people with dementia and the growing importance of PC as a hub of care [1,15], it is important to understand more about how PC provides care to people with dementia and their family caregivers [1,13,16] and how this care is perceived in relation to their needs and expectations. To address this gap, the present study explores the experiences of people with dementia and their family caregivers during the period before, during, and after the dementia diagnosis, with particular attention to the care provided and the support received. Understanding these experiences is crucial for identifying strengths and shortcomings in current care pathways and informing improvements in dementia care delivery.

Box 1. The role of primary care in Swedish health care and in the process of dementia assessment.

Primary care in Sweden is organized by the regional health authorities and operates separately from the municipalities, which are responsible for providing social care services [17]. An increasing amount of care is being shifted from hospitals and outpatient specialist services to primary care. This shift places significant responsibility on primary care providers for the diagnosis and follow-up of a wide range of conditions [18].

According to the *National Guidelines for Care and Support in Dementia Disease* issued by the Swedish National Board of Health and Welfare [8] the assessment of suspected dementia involves several health care professionals situated in primary and outpatient specialist care, and should be structured according to the following stages:

1. The **basic assessment in primary care** should include a blood sample, cognitive tests (mini mental state, clock drawing test), an evaluation of the person´s functional and activity abilities, as well as a structural brain imaging with, e.g. computed tomography.
2. Usually, the ICD-10 code for dementia is not assigned in primary care; instead, the patient is referred to a **specialist memory clinic** for an in-depth evaluation and possibly a lumbar puncture. The memory clinic establishes diagnosis, creates a care plan, and may initiate medication.
3. After having received the diagnosis, the person with dementia should be followed up at least once per year in health care. It is not specified which care instance should **follow up** but in practice this often happens in primary care. The follow-up should include a medication review, a structured assessment of the person’s functional and activity abilities, conversations with relatives, and an update of the person´s care plan.

Of note, rural areas in Sweden may lack memory clinics resulting in a more prominent role of primary care during the entire process.

## Method

### Study design

This qualitative study draws on the lived experiences of people with a diagnosis of dementia and their family caregivers. We used a constructivist framework for the analysis with an understanding of knowledge as constructed through individual lived experiences and awareness that our lived experiences as researchers influence knowledge that we generate in interaction with our research subjects and data [19]. This approach requires researchers to embrace the narratives of informants without introducing preconceptions or comparisons with established facts. Data collection consisted of semi-structured interviews [20], and data analysis was based on an inductive thematic approach [21].

### Participants and recruitment

People with dementia diagnoses and varying stages of dementia were recruited based on the following inclusion criteria: aged 65 years or older, able to speak Swedish or English, capable of conversation, living at home and having the capacity to consent to participation. To assess the capacity to consent to participation for people with a dementia diagnosis, we first asked the person directly whether they wished to take part in the interview. This conversation took place together with the family caregiver who was familiar with the person’s cognitive status and communication abilities. The family caregivers were adult children or partners to a person diagnosed with dementia.

All informants were recruited in collaboration with the Dementia Association which is an organization that works to support and promote the interests of people living with dementia and their relatives (www.demensforbundet.se). By providing information, advice, and support, the organization strives to raise awareness about dementia and improve the quality of life for those living with the disease. In the recruitment process the organization sent out information about the research project through their channels, both digital and physical, to persons diagnosed with dementia and their relatives. People who were interested could then contact the research group for further information. Seventeen people contacted the research team and 15 were interviewed, four persons with dementia and 11 family caregivers (see Table 1). The reason for those two interested people not participating was that one could not be reached for further planning, and the other one was hospitalized.

**Table 1.** Characteristics of informants (*n* = 15).

Variables	People diagnosed with dementia	Family caregivers
Gender		
Male	3	4
Female	1	7
Age (years)		
Mean	79	75
Range	74–87	55–87
Education		
Pre-secondary and secondary	2	3
University	2	8
Born		
Sweden	4	9
Abroad	–	2

### Data collection

Semi-structured interviews [20] were conducted between February and April 2024 by two of the authors (MB and PBR). All informants lived within the geographical area of the Stockholm region and were interviewed face-to-face in their homes, or in facilities at the Stockholm Region or the Dementia Association. All people diagnosed with dementia were interviewed together with a family caregiver to ensure that they felt comfortable and supported throughout the interview.

Based on the aim of the study, an interview guide with predefined topics was prepared to initiate and structure the interviews. To cover different aspects of how to improve care the interviews started with broad open-ended questions, while the probing questions differed to give the informants the opportunity to elaborate and dwell on the aspects they chose as most relevant. Each answer was followed up by further probes, such as ‘Can you explain this further?’ or ‘Could you please give an example?’ Questions included were: (1) Can you describe what occurred when you/your relative received the dementia diagnosis? (2) Who is the primary caregiver for you/your relative, and what does their care routine involve? and (3) What kind of help or support do you currently receive, and what additional support do you feel is lacking?

Data collection continued until information power was reached [19], i.e. when sufficient data had been collected to develop a robust and valid understanding of the aim. Using the concept of information power, we determined our aim to be narrow, and that dense sample specificity could be achieved by recruiting informants with substantial experience in the issue, supporting a lower sample size. Therefore, we considered 15 respondents to be adequate to address our research objectives. All interviews were audio-recorded and transcribed verbatim. The interviews ranged between 56 and 92 min with an average length of 71 min.

### Data analysis

The transcribed interviews were analyzed using a thematic analysis, a method for identifying, examining, and conveying patterns of significance within the data [21]. The analysis was carried out manually. First, all authors read the transcripts several times to grasp the entire material. The data set was organized by coding all text into meaningful units. Initially, two qualitative researchers (MB and PBR) separately coded and collated codes with similar content into potential subthemes. These authors discussed how potential subthemes were related by similar content or processes, and generated potential themes. When opinions differed, parts of the transcripts were reread. The researchers reviewed potential subthemes during repeated discussions and merged subthemes considered to have a common origin, constructed refined themes, and identified illustrative quotes in the data. Finally, all nine sub-themes were linked to four overarching themes (see Table 2). The other researchers (KM and KSM) conducted reciprocal reading between transcripts and themes/subthemes to ensure no data was overlooked, to further refine the name and definition of each theme.

**Table 2.** Overview of the themes and Sub-themes related to how informants with dementia and family caregivers experienced the period prior, during and after diagnosis and what kind of support is needed.

Themes	Sub-themes
Seamless assessment from primary care to memory clinic	Empathy and professionalism are crucial to prevent feelings of failure Diagnostic disclosure – both shock and sorrow, yet also relief
After diagnosis – a no man’s land	Gap of silence between memory clinic and primary care Feeling of being left behind causes emotional distress and isolation
Follow-up in a deficient primary care	‘A single point of entry’ for access to coordinated care in a fragmented care system Continuity, expertise and knowledge in dementia care is crucial yet often missing
Embracing life with the diagnosis	Need for guidance by a dedicated dementia team to manage the progressive nature of the disease Lack of support for the person with dementia Finding the way without a map

### Trustworthiness

The research team consisted of people from different backgrounds, such as behavioral science, epidemiology, general practice, and nursing, all contributing unique perspectives. The authors have a wide range of experience in research and clinical practice in eldercare, as well as a broader perspective on health services. To ensure credibility, the research team followed the Braun and Clarke’s guidelines [21] and the Consolidated Criteria for Reporting Qualitative Research (COREQ in supplementary file) [22]. Confirmability was strengthened by using quotes from the collected data. These were translated from Swedish to English by the researchers. Throughout the analytical process, discussions were held with all authors to strengthen the credibility of the findings. To further strengthen the study’s credibility, the research team collaborated with a reference group from the Swedish Dementia Association (www.demensförbundet.se). The group’s role was to provide input on the study design, interview guide, and the credibility of preliminary results. The reference group included association staff, persons living with dementia, and relatives.

### Ethical considerations

Before the interview, informants who agreed to participate received oral and written information about the aim of the study and how the study results should be used. Furthermore, they were informed several times that they could decline further participation at any time during the interview and that their participation was confidential, i.e. all names and places were deleted in the transcript. The informants gave their oral consent during the recruitment process and before the interview started. This consideration was made because dementia is by nature a memory disorder; therefore, the first consent cannot be considered valid on a later occasion [23]. Moreover, during the interviews, the interviewer observed non-verbal signs of inconvenience or indications of wishes to withdraw from the interviews [24]. If any such expression had occurred, the researcher would have interrupted the interview. However, no such situation occurred. The Regional Ethical Review Board in Sweden, (Reg. no. 2023-05475-01) approved the study.

## Results

The analysis distinguished four themes that reflected the experiences of informants with dementia and family caregivers prior, during and after the diagnosis, along with their needs for support. The first theme *Seamless assessment – from primary care to memory clinic* described the transition from PC to memory clinics, where empathy and professionalism were crucial to prevent feelings of failure. Receiving a diagnosis gave rise to contradictory emotions, including shock, sorrow, but also relief. The second theme *After diagnosis – a no man’s land* highlighted a communication gap between memory clinics and PC, leaving informants feeling abandoned and causing emotional distress and isolation. In the third theme *Follow-up in a deficient primary care*, navigating a disjointed care system underscored the importance of having ‘a single point of entry’ for coordinated support. A lack of continuity and expertise in PC often exacerbated difficulties. The final theme *Embracing life with the diagnosis* emphasized the need for comprehensive guidance from a dedicated dementia team to manage disease progression. Informants with dementia and their family caregivers valued personal connections that affirmed their individuality and dignity, likewise, understanding what to expect in the future remained vital for planning and adapting to their evolving situation.

### Seamless assessment – from primary care to memory clinic

#### Empathy and professionalism are crucial to prevent feelings of failure

All informants with a suspected memory disorder initially underwent a basic assessment at their PC practice, followed by a referral for an in-depth diagnostic evaluation with a specialized cognitive team at a memory clinic. They described the initial assessment in PC as prompt, efficient, and professional, and they were rapidly given appointments in memory clinics without prolonged waiting period. Both the persons being assessed, and their family caregivers reported that the team′s conduct during the extended evaluation made them feel well cared for, with an atmosphere of empathy and respect. One informant emphasized that the staff was approachable, warm, and understanding, which significantly alleviated the persons anxiety and mitigated feelings of inadequacy during the diagnostic procedures.

*Person diagnosed: It is important to feel that you haven’t failed (during the tests). So even if that were the case, you don’t need it spelled out for you.*

*Interviewer: So, you said empathy is important and that one shouldn’t feel stupid.*

*Person diagnosed: No, not failed and stupid. When they took the spinal cord test, it was the same atmosphere - positive and light. I got a lot of praise when I managed to sit in the position that was required and so on. It was a pleasant meeting in the middle of everything.*

#### Diagnostic disclosure – both shock and sorrow, yet also a relief

Many times, the person with memory disorder and family caregivers suspected dementia before a formal diagnosis, based on symptoms and heredity. However, receiving the diagnosis still came as a shock, leaving them unsure of how to cope with the news. Family caregivers described an initial reaction of shock and denial, followed by feelings of sadness and a sense of unreality. One family caregiver described that receiving the diagnosis felt like a relief as their suspicions were confirmed.

*Family caregiver: Yes, I think we just… went black… both of us, really. And we just went silent. And I just cried. And then…I mean, it can’t be true. And when you get it this early, you can usually live a very good life for a few years (said the memory clinic). That doesn’t comfort me at that point, though.*

*Family caregiver: We walked away with a diagnosis, but we were very calm because we already knew how things stood.*

### After diagnosis – a no man’s land

#### Gap of silence between memory clinic and primary care

After receiving a dementia diagnosis from the memory clinic informants felt abruptly left alone. Besides a written referral to PC for follow-up, only minimal information or guidance on how to move forward were offered. The delay until follow-up varied depending on the PC practice they attended. Only a minority were given an appointment within 6–12 months from the referral. Instead, several informants had to reach out to the PC provider themselves after a year which often resulted in feelings of being abandoned in a ‘gap’ between the memory clinic and PC. Family caregivers’ expressed frustration over the lack of support and felt burdened by the responsibility to ensure necessary care for the person with dementia.

*Family caregiver: And it was said at the memory clinic that now we’ll send all the papers to your primary care clinic and then they’ll take over. But we kind of thought they would call us. But nothing happened, instead, you had to make contact yourself. So, I would say that some kind of follow-up is definitely missing here.*

#### Feeling of being left behind causes emotional distress and isolation

Once diagnosed with an incurable disease, it felt as if health care disregarded both the person with dementia and family caregivers. They felt overlooked and dismissed, as though no one truly cared or took their situation seriously. At the same time, they had many questions such as ‘What will happen now? Will we be able to manage on our own? Who may help us?’ but no one to turn to for answers. The sudden feeling of being left alone left them unsure of what to do next or whom to contact.

*Family caregiver: But it’s a sad thing that nobody contacted us, and we didn’t know what to do next. She (the mother) didn’t feel like anyone took her seriously and no one cared for her. Plus, she didn’t really understand herself that it was a pure memory-related disease she had been diagnosed with.*

### Follow-up in a deficient primary care

#### ‘A single point of entry’ for access to coordinated care

A key challenge faced by all informants after diagnosis was the difficulty of navigating in a complex health care system after minimal guidance from the memory clinic. They expressed frustration as they struggled to understand who was responsible for what in health care and municipality, e.g. with reference to follow-up after diagnosis and prescription of medications. Family caregivers desired streamlined access to care with ‘a single point of entry’ to avoid multiple providers and procedures. Lack of information sharing, insufficient coordination and communication between municipalities and health care regions made their situation even more challenging. Family caregivers emphasized the need for a liable contact person, a ‘spider in the web’ offering guidance on available services and benefits across both municipality and health care region.

*Family caregiver: It’s a bit difficult walk out of there and know where to start looking for information. I don’t really understand health care system at all. And then we all have to start searching because nothing is coordinated. Who should call? Who takes care of this? … It is just expected that the relatives will sort it out. I would have wished it were much easier to get in touch with health care, more like a ‘a single point of entry’, because there are so many steps that are quite hard to navigate.*

#### Continuity, expertise and knowledge in dementia care is crucial yet often missing

Both the person with dementia and family caregivers preferred to have a regular GP contact. Those who had had contact with the same GP for a longer period of time emphasized the benefits of continuity, security, and open communication. GPs who were familiar with their patients were described as more attentive, taking their problems seriously, and providing guidance on various issues, including home care and community services.

*Family caregiver: And especially the fact that she’s been with us the whole time (the GP). She always listens to us. And she takes us seriously. I don’t have to retell my story every time I meet her because she knows everything about us. It’s fantastic to have a doctor like that. Yes, it’s such a comfort… we both have her so it’s really reassuring.*

Informants with dementia who lacked an assigned GP often felt dissatisfied with their follow-up in PC. They reported that PC fell short in providing an individualized care plan or in structuring the contents of a follow-up appointment. Additionally, informants felt that these visits were detached and unengaging. For example, the effects of dementia medication were not thoroughly evaluated, and while blood samples might be taken, the results rarely influenced medication dosages. This created uncertainty about the appropriateness of treatment. Family caregivers noted that the GP were unaware of the memory clinic’s medical record, and each of the person’s comorbidities were addressed separately, highlighting the fragmentation of health care, with no one taking overall responsibility for care.

*Family caregiver: When you come to these follow-up appointments, it’s like… no one is really interested … instead they ask why are you here?*

*Person diagnosed: They don’t know what to do. No, that’s how it feels. They don’t know how to start treating me.*

*Family caregiver: They don’t seem to have a plan for following up with someone who has a dementia diagnosis.*

*Person diagnosed: You think it’s just me who has this. But it’s not. …It feels very lonely. And they don’t have time for that. …That’s how I feel.*

*Family caregiver:. They run a few tests. It feels like they’re only doing it because we’ve asked for an annual check-up. And they renew the prescriptions just to get rid of us….*

In absence of both a structured follow-up and a care plan from PC, the informants with dementia and family caregivers experienced that PC lacked expertise and commitment for them as individuals and for dementia in general. Many family caregivers had questions about behavioral changes. Still, as GPs tended to focus on physical conditions and their treatment, they did not receive the support or advice they needed, such as practical tips regarding how to cope with living with dementia.

*Family caregiver: After dementia was confirmed, we’ve only had contact with the general practitioner. He has also told me that he can only check the physical aspects. He said, ‘I have nothing to do with the dementia evaluation’, but he can see the deterioration. ‘I can check her physically to make sure she hasn’t hurt herself or prescribe sleeping pills if needed. But I don’t handle this illness, that’s the responsibility of those who ­diagnosed it.*

*Interviewer: But you said you are not welcome there anymore.*

*Family caregiver: No, there’s a gap.*

Family caregivers described that they were expected to coordinate the entire care process for the person with dementia. According to one family caregiver, PC was not receptive to her wishes for a different, ‘more compassionate’ type of care. She explained how she was able to calm her husband using the power of music but wanted additional guidance which PC could not provide her with.

*Family caregiver: She (the doctor) wasn’t really prepared to listen to my questions. My husband was in a very advanced stage, so I wanted to know what more I could do to stimulate him besides music. ‘Yes, the medication’, the doctor replied. She didn’t listen when I said I didn’t want him to receive a lot of anxiety-relieving medication. But the doctor here at the primary care wasn’t really someone who listened; she just said no, he’s too far gone.*

### Embracing life with the diagnosis

#### Need for guidance by a dedicated dementia team to manage the progressive nature of the disease

Family caregivers and the informant diagnosed expressed a substantial need to discuss the disease with someone who has medical expertise about dementia and at the same time could guide them through its various stages and behavioral challenges. Currently, the type of support they received varied greatly and PC was perceived as ‘mechanically’ following up the disease, often lacking both the interest and knowledge to offer comprehensive care. Municipalities were described as more accessible than regional health care services, but still, informants experienced that they lacked expertise in dementia care. Municipalities organize meetings for family caregivers which they evaluated as valuable but insufficient. In some municipalities, a dementia nurse offered advice and general information but no tailored guidance.

Both the informants with dementia and their family caregivers expressed a need for more personalized care in PC and municipality as each person’s needs go beyond standard dementia guidelines. Informants propose a centralized PC unit which should be specialized in dementia care, or a team linked to the memory clinic. Such a unit should comprise a specialized dementia care team to provide ongoing, individualized treatment and follow-up similar to the rehabilitation at occurrence of other conditions, e.g. diabetes or breast cancer.

*Family caregiver: This illness lasts for a long time. And if you don’t know yourself how to manage it, then there needs to be information about it, presented in a way that’s a bit more personally engaging. I can read on websites, through links, and in all kinds of places and books. But it’s still not… I would like there to be a unit, like there is for habilitation, but instead, it would be a team for dementia… a specialist health center with follow-up care. You’d want it to be a cohesive system in health care.*

#### Lack of support for the person with dementia

Informants expressed that there is limited support for the person with dementia, despite need for targeted assistance. After a diagnosis, all informants considered that the available support focused primarily on family caregivers rather than the person with dementia. While some family caregivers were offered counseling and information about dementia through lectures and courses, the informants with dementia were solely offered a place at the municipality’s day care center. However, in the early phase of dementia the day care center may not be suitable as it targets people with more severe dementia symptoms. In the current system with insufficient support, the informants with dementia felt abandoned, with their family caregivers being their only source of support. They sought continued contact with a nurse or doctor they trusted, who knew them personally and had expertise in dementia, and could provide support.

*Family caregiver: There is a carer coordinator (in the municipality) since a few years. And there is support for the elderly and people with disabilities and …*

*Person diagnosed: It’s for carers not for me.*

*Family caregiver: We advise each other in this carers group. But it is a support for me. There is nothing for … There is no support for X (the person with dementia). Nothing at all.*

#### Finding the way without a map

Due to the lack of practical information and support from health care services, family caregivers were often frustrated as they must educate themselves about dementia through personal research, attending lectures, and reading blogs. They emphasized the importance of receiving proper guidance to manage the progressive nature of the disease, felt that each family was left to ‘reinvent the wheel’ after a dementia diagnosis. They highlighted the need for a simple, clear checklist with practical advice on what to do after having received the diagnosis. They desired a leaflet that outlines the various types of support and services available from both health care providers and municipalities. This information should be displayed in all PC practices and memory clinics.

*Family caregiver: And this sad thing that most of us in the relatives’ group have said. That it’s so strange that every single family has to reinvent the wheel. That you can’t just get a folder with a checklist or something. It creates so much unnecessary work for everyone.*

Some family caregivers had been offered counseling through PC, still, they questioned its benefit as they rather sought concrete support in managing both the relationship with the person diagnosed and the challenges surrounding their care. They expressed a need for more specialized psychiatric and psychological support and the importance of trained professionals who understand their family situation, and whom they trust. For example, they wanted to talk about coping without knowing what the future holds, the changing relationship with the person with dementia, and the guilt, anger and sadness they felt in relation to the person’s dementia. They requested concrete guidance on how to handle emotional and existential concerns, such as how to manage personality and behavioral changes in dementia and how to plan for the future. Having a clear plan and vision for what lies ahead was crucial, especially during emotionally challenging phases. Family caregivers sought support in finding meaning in life despite the diagnosis, so they would not become overwhelmed by a sense of hopelessness. While support groups with others in similar situations were beneficial for some, others found it difficult to discuss the person with dementia in such settings, as it might feel like a betrayal of the person’s privacy.

*Family caregiver: I have called the health center to speak with a psychologist. He didn’t understand anything. He said, ‘I’ll talk to my colleagues and get back to you’. He never got back to me.*

*Interviewer: What do you wish he had done for you?*

*Family caregiver: Well, for example, give me advice on how to handle this situation.*

*Interviewer: What is it that you need to handle?*

*Family caregiver: I need to handle life somehow. What are we supposed to do in the future?*

## Discussion

In the early stages of dementia assessments conducted in PC and memory clinics, both informants with dementia and family caregivers reported experiencing treatment characterized by knowledge and respect. (*Theme 1*). However, challenges emerged when PC was expected to take over the responsibility for follow-up after the person had been diagnosed at the memory clinic (*Theme 2*). Here, informants described feeling abandoned, as the memory clinics did not give adequate guidance, and PC did not offer any follow-up appointments. This transitional ‘gap of silence’ in care trajectories was associated with emotional distress and feelings of neglect.

Family caregivers experienced that the lack of a follow-up after a diagnosis often forced them to take the initiative in arranging follow-up appointments in PC and managing the renewal of prescriptions, resulting in frustration and an overwhelming sense of responsibility for the care. In the literature, navigating each phase of the diagnostic process of dementia is experienced as ‘living with uncertainty’ by patients and their families describing a need for information and support at all phases in the disease trajectory [25]. This is aligned with our findings as many informants describe how they frequently struggled to find information and support, particularly when confronted with unanswered questions about the course of disease and future care needs (*Theme 3*).

Furthermore, the informants in this study reported difficulties navigating a complex care system with unclear divisions of responsibility between health care providers as well as between the regional health care and municipality care. They emphasized the need for ‘a single point of entry’ to access comprehensive, coordinated care (*Theme 3*). The ability to have a single point of contact, as opposed to having to contact numerous services was highly valued among our informants and are in line with earlier research [12,26]. There are experiences from memory clinics across Europe where individuals diagnosed with dementia are connected to support through ‘a single point of entry’, sometimes referred to as a ‘single service’ model, ‘link worker’, ‘admiral nurse’, ‘dementia advisor’, a ‘care navigator’ or ‘case manager’ [27,28]. A multi-country European study, including Sweden, emphasized the value of a designated contact person—such as a nurse or key coordinator—for people with dementia and their informal caregivers, easing communication and improving continuity of care [29]. Similarly, an Australian study highlighted the importance of having a single point of post-diagnostic support, noting that while the role may vary, its core function is to make support more accessible after diagnosis [30]. While models that include a key support person can look different, they all share the idea that this person plays a central role in making support accessible after a dementia diagnosis. When care systems lack an integrated way of working, persons diagnosed and family caregivers perceive their care as fragmented, with no single entity assuming responsibility for coordinating the person’s care. Consequently, a significant administrative burden is placed on them [9,31] which undermines trust, confidence, and continuity of care [6,9]. As a result, family caregivers need to coordinate care [8,12].

Many informants expressed the need for PC professionals to improve their knowledge and skills in dementia care (*Theme 3*). They perceived GPs as lacking knowledge about dementia, which often hindered them contributing constructively to the care process. Informants interpreted the absence of follow-up on the effectiveness of dementia medications, or the failure to address questions about disease progression and existential concerns as indications of inadequate expertise. The lack of such individually tailored counseling contributed to uncertainty regarding the treatment approach. This is in line with earlier research where people diagnosed with dementia report that many health care services are seen as overly focused on the medical aspects of the disease, while psychological and social needs are frequently overlooked or insufficiently addressed [12,32].

In the current study, informants expressed a wish to maintain contact and receive follow-up from their GP after receiving a dementia diagnosis. Previous research has shown that effective post-diagnostic GP follow-up relies on a trust-based relationship characterized by mutual understanding, personal familiarity with the person with dementia, empathy, supportive care, and respectful communication [33]. However, both people with dementia and GPs have reported that contact is too infrequent to build or maintain a strong relationship. Notably, people with dementia tend to value their relationship with their GP even more after having received a dementia diagnosis, underscoring the importance of strengthening this continuity in dementia care [34]. Given the high prevalence of cognitive decline [35], dementia care should play a more prominent role in the education of GP residents and medical students, as current training remains insufficient [36].

Informants in our study expressed a need for more specialized psychiatric and psychological support, as well as practical advice on how to handle future behavioral changes. Previous research indicates that GPs often lack sufficient knowledge about how to manage personality and behavioral changes associated with dementia. Moreover, they experience the diagnostic process overwhelming and beyond their expertise [37]. Also, in the same study, GPs reported that their support was often limited to prescribing sedative medications and that the lack of comprehensive clinical guidelines in this area presented substantial challenges [37]. Given the aging population and the increasing prevalence of dementia, specialist knowledge in dementia care should be integrated into PC practice. Furthermore, effective communication training for health care professionals is essential to ensure consistent, empathetic, and well-informed interactions with patients and family caregivers [6].

Both family caregivers and informants with dementia request more support from health care and municipality, especially with questions about disease progression and existential concerns (*Theme 4*). All informants express that current care offers insufficient guidance and long-term planning leading to feelings of anxiety and uncertainty about what lies ahead. Instead, informants suggest that integrated follow-up of dementia should be done by a specialized team, like the teams available in cancer care. Such teams include a dedicated liaison nurse who plays a vital role as a consistent point of support for the person with dementia and their families throughout the course of the disease. These teams may also answer questions about how to manage personality and behavioral changes, mental and emotional health and activities of daily living, i.e. meeting the needs of people with dementia and family caregivers [9]. This is in line with previous research which describes that a multidisciplinary team with professionals from both the municipality and the health care region surrounding the person diagnosed and their families contribute to better coordination, continuity and increased efficiency [8,9,16]. Our findings and earlier studies suggest a need for a more integrated model of care for dementia, focusing on continuity, specialized support and better communication between care providers [6,8,9].

Informants diagnosed with dementia in this study describe how they lack individually tailored support in the beginning of their disease course from both health care and municipality (*Theme 4*). Besides day care, the municipality does not offer much support for them besides day care which aims at individuals with advanced dementia symptoms rather than those with mild symptoms. Moreover, informants feel that neither the municipality nor PC recognize them as unique individuals with capabilities and capacity to make their own decisions although in need of support. This aligns with former research highlighting that people with dementia want to be treated as a person that should be informed and actively involved in decisions [38]. Additionally, people with dementia seek opportunities to better understand their disease by connecting with others in similar situations as they value hearing firsthand accounts of experiences, learning what to expect, and discovering effective coping strategies [39]. Similarly, family caregivers in this study have varied experience with the municipalities′ support groups but emphasize the importance of having opportunities to speak with professionals with expertise in dementia and understanding the family situation.

## Strengths and limitations

Patient involvement is a crucial component of dementia research priority-setting exercises, ensuring that research are relevant and acceptable to those who stand to benefit the most [16]. However, the inclusion of people living with dementia in research remains a somewhat neglected area due to the challenges associated with engaging people experiencing cognitive decline [40]. Our findings revealed significant alignment between the perspectives of people with dementia and family caregivers. Involving both groups provided a more comprehensive and nuanced understanding of the needs of individuals living with dementia and their families.

The informants with dementia were generally more reserved than their family caregivers, resulting in much of the information in this study coming from the caregivers, despite efforts to specifically engage the person with dementia. The interviewers sometimes found it difficult to get the person with dementia to talk because the family caregivers might answer for the person. On these occasions, the interviewer repeated the question, specifically addressing the person with dementia. However, in general the four informants with a dementia diagnosis spoke openly from their perspective.

Thematic analysis of individual interviews is appropriate when aiming to find a pattern of meaning across a data set, as in our study [21]. The analysis was enriched by multiple researchers with different experiences and competencies from the medical, nursing and sociological fields, and by two coders who discussed similarities and differences in the coding process as part of theme generation. It is possible that the analysis would have been further enriched by repeated interviews, but this was beyond the scope of the project. The research group has presented the interview results to the reference group at the Dementia Association (www.demensförbundet.se) to ensure that all relevant perspectives were considered. Transparent reporting of methodological processes allows readers to identify transferability of our findings in other settings.

## Conclusion

Both persons with dementia and their family caregivers in the present study reported that primary care often falls short in meeting their needs, mentioning limited accessibility, fragmented services, and a lack of dementia-specific competence among care providers. To address these gaps, integrated and individualized care must be prioritized throughout the course of the disease. First, GPs often serve as the first point of contact and play a key role in ensuring continuity, coordination, and person-centered care, making it essential to strengthen their role. Moreover, dementia care should be given greater emphasis in medical education and residency training. Second, introducing liaison nurses within dementia care who collaborates closely with both GP and a municipal contact person may facilitate care coordination, simplify navigation of the health care system, and offer consistent support to persons with dementia and their family caregivers. Third, a holistic approach in primary care may bridge information gaps, reduce anxiety, increase the sense of security, and improve overall health and well-being. Our findings underscore the need for health care policy in Sweden to promote sustainable dementia care models that reinforce the GP’s role and support person-centered, multidisciplinary primary care.

## Supplementary Material

Supplementary file COREQ.docx

## Data Availability

The interview data that support the findings of this study are not publicly available due to them containing potentially identifying or sensitive information that could compromise the privacy of the research informants.
